# Regulatory T cells in autoimmune diseases: biological mechanisms, clinical translation, and translational challenges

**DOI:** 10.3389/fimmu.2026.1881360

**Published:** 2026-08-13

**Authors:** Jingchang Li, Jia Pang, Peipei Wu, Enmei Wang, Shijun J. Zheng, Qingguo Ruan, Ruiling Liu

**Affiliations:** 1 Joint National Laboratory for Antibody Drug Engineering, Henan University School of Medicine, Kaifeng, China; 2 School of Basic Medical Sciences, Henan University School of Medicine, Kaifeng, China; 3 Eye Institute of Shandong First Medical University, Qingdao, China

**Keywords:** autoantigen, autoimmune disease, immune tolerance, immunotherapy, regulatory T cell

## Abstract

Autoimmune diseases (ADs) are a group of inflammatory disorders triggered by aberrant immune responses against autoantigens and the breakdown of immune tolerance. Current therapeutic strategies, such as glucocorticoids and immunosuppressants, exert largely non-specific immunomodulation and are frequently associated with substantial adverse effects upon long-term administration. As such, these approaches are often insufficient to re-establish immune homeostasis. In recent years, regulatory T cell (Treg)-based therapeutics have undergone rapid advancement, with continuously updated therapeutic modalities broadening the application boundary of immune tolerance intervention. This review comprehensively outlines the progress of Treg-based therapeutics, spanning from fundamental biological research to translational applications in autoimmune diseases. We describe the developmental characteristics, suppressive machinery, and heterogeneity of Tregs, and highlight the preclinical and clinical advancements of diverse Treg therapeutic modalities, including polyclonal and antigen-specific Tregs, TCR-engineered Tregs, and CAR-Tregs. Furthermore, we emphasize current optimization strategies for enhancing Treg stability, antigen specificity, and *in vivo* fitness within inflammatory microenvironments. Additionally, we objectively address the major translational bottlenecks of Treg therapy and provide future perspectives to facilitate the clinical implementation of Treg-based immunotherapies for autoimmune diseases.

## 1 Introduction

**Figure 1:**
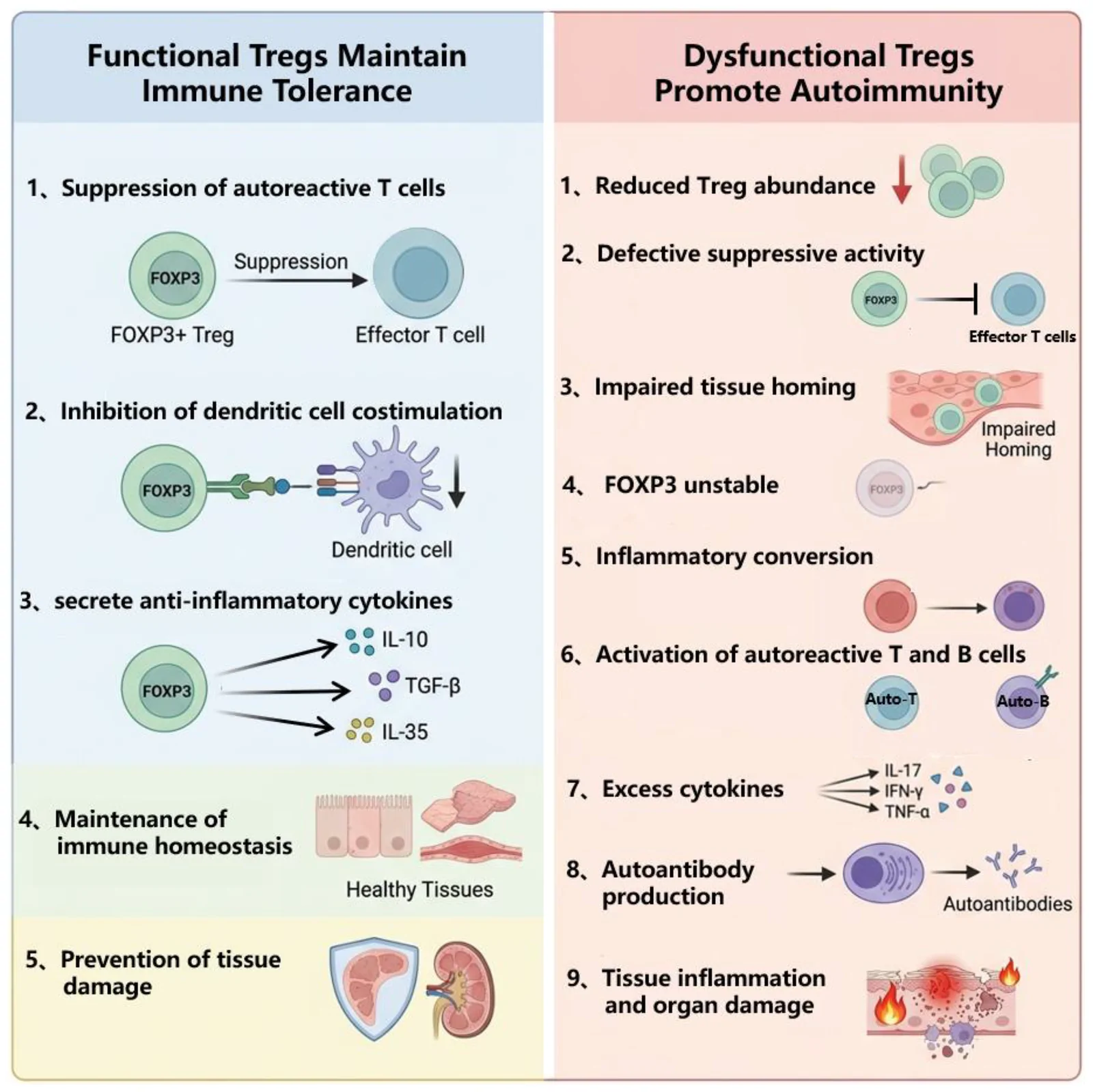
Schematic overview of Treg dysfunction in autoimmune disease pathogenesis. Under physiological conditions, Foxp3^+^ Tregs maintain peripheral immune tolerance by suppressing autoreactive T cells, restraining antigen-presenting cell activation, consuming IL-2, and producing anti-inflammatory mediators such as IL-10, TGF-β, and IL-35. In autoimmune diseases, Treg-mediated immune regulation may be disrupted through reduced cell number, impaired suppressive function, lineage instability, defective tissue homing, and inflammatory conversion. These abnormalities contribute to hyperactivation of autoreactive T and B cells, increased inflammatory cytokine production, autoantibody generation, chronic tissue inflammation, and progressive organ damage.

Autoimmune diseases (ADs) are a group of chronic inflammatory disorders caused by aberrant immune responses against autoantigens and the breakdown of immune tolerance. Their pathogenesis is highly complex, with immune dysregulation representing a central pathogenic mechanism, particularly the disruption of peripheral tolerance maintained by regulatory T cells (Tregs) (1). As a key regulatory cell subset for the maintenance of immune tolerance, abnormalities in the number or function of Tregs can lead to the erroneous recognition of autoantigens by autoreactive immune cells and subsequent autoimmune attack, thereby triggering the onset of ADs (2). **Figure 1** schematically illustrates the dysfunction of Tregs during the pathogenesis of autoimmune diseases.

ADs can affect virtually all organs throughout the body, encompassing both systemic disorders, such as systemic lupus erythematosus and rheumatoid arthritis, and organ-specific diseases, including autoimmune thyroid disease, type 1 diabetes, and inflammatory bowel disease. These conditions commonly lead to chronic inflammation, tissue damage, organ failure, and even functional disability. In most cases, they follow a prolonged clinical course, are difficult to cure, and therefore require long-term standardized management. Conventional therapies still suffer from considerable limitations. Taking systemic lupus erythematosus (SLE) as an example, this autoreactive B cell-driven autoimmune disease remains primarily managed with glucocorticoids as first-line therapy, which non-specifically suppress systemic inflammatory responses (3). Other conventional treatment options include traditional immunosuppressants (e.g., cyclosporine), targeted biologic agents (e.g., rituximab and belimumab), antimetabolites (e.g., azathioprine and methotrexate), and alkylating agents (e.g., cyclophosphamide). However, these therapeutic approaches generally share major shortcomings, including limited specificity and a failure to reverse the underlying pathogenesis (4). Quantitative data highlight the unmet need. In RA, approximately 50% of patients fail to achieve an optimal clinical outcome or experience adverse reactions following methotrexate treatment (5). A study involving 1,069 patients showed that 77.5% of subjects experienced at least one methotrexate-related adverse event (5). In SLE, 45–60% of patients relapse within 17 months after receiving rituximab treatment (6).

Compared with conventional therapies, cell-based therapeutics represented by Tregs offer not only greater therapeutic specificity but also the potential to cure ADs by re-establishing immune tolerance (7). Given that Tregs are the core cellular subset responsible for maintaining peripheral immune tolerance, this review provides an overview of the association of the number and function of Tregs with the pathogenesis of ADs, elucidates the molecular mechanisms by which Treg dysfunction disrupts immune homeostasis, and discusses clinical translation and future prospects of Treg-based immunotherapeutic strategies.

## 2 Discovery, classification, and functions of Tregs

### 2.1 Discovery of Tregs

Tregs represent a major subset of CD4^+^ T cells, and their research history can be traced back to the 1970s and 1980s, when multiple rodent studies had already suggested the existence of a T-cell population capable of suppressing the development of experimental autoimmune diseases. In 1995, Sakaguchi et al. demonstrated that CD4^+^CD25^+^ T cells are indispensable for maintaining peripheral tolerance (8–10). In addition to Sakaguchi’s pioneering work, other investigators including Ethan Shevach, Fred Ramsdell, Alexander Rudensky, and Jean-François Bach made foundational contributions to Treg biology (11). Collectively, these findings established the indispensable role of CD4^+^CD25^+^ T cells in the maintenance of peripheral immune tolerance, and this population was subsequently designated as Tregs.

Human CD4^+^CD25^+^ Tregs were identified in 2000–2002 (12). In 2001, Foxp3 mutations were linked to IPEX (13); in 2003, Foxp3 was identified as the master transcription factor for Tregs (14–16), and TGF-β was shown to induce Foxp3 expression (17). Notably, the pioneering contributions of these scientists to the understanding of peripheral immune tolerance were recognized with the 2025 Nobel Prize in Physiology or Medicine (Nobel Assembly at Karolinska Institute, 2025).

### 2.2 Classification and functions of Tregs

Tregs display well-defined heterogeneity and can be classified into distinct subsets according to their developmental origin and tissue distribution, with each subset exhibiting unique biological characteristics. Based on developmental origin, Tregs are generally divided into three major subsets that can be distinguished by specific markers: thymus-derived Tregs (tTregs), which constitute the principal subset and arise during thymic development; peripherally derived Tregs (pTregs), which differentiate extrathymically within peripheral tissues; and induced Tregs (iTregs), which can be generated *in vitro* under TGF-β-driven culture conditions (18).

Among these subsets, the Ikaros family transcription factor Helios has been proposed as a specific marker for tTregs. Naturally developed tTregs stably express high levels of Helios, whereas pTreg and iTregs show little or no expression of this molecule (19). In addition, Helios^+^ Tregs exhibit a higher degree of demethylation within the Treg-specific demethylated regions (TSDRs) of the Foxp3 locus, indicating greater lineage stability (20). Compared with neuropilin-1 (Nrp1), another candidate marker of Tregs, Helios appears to provide superior specificity for discriminating Treg subsets (21), thereby offering important experimental support for precise Treg identification and quality control in Treg-based therapy.

From the perspective of tissue distribution, Tregs can further differentiate into tissue-adapted subsets specialized for distinct anatomical niches, each characterized by unique molecular signatures and functional properties. **Table 1** outlines the origin, markers, tissue distribution, and functional characteristics of different Treg subsets. Recently characterized markers encompass ST2 for adipose-resident Tregs, RORγt for intestinal Tregs, as well as CD38, CD39 and LAG-3, which serve as indicators for Treg activation and stability (22). Adipose tissue-resident Tregs highly express PPARγ and ST2 and primarily regulate adipose inflammation and metabolic homeostasis (23, 24). Intestinal resident Tregs express RORγt and are essential for maintaining mucosal immune tolerance in the gut (25). Tumor-infiltrating Tregs are characterized by high expression of ICOS, PD-1, and CCR8, and promote tumor progression by facilitating immune evasion. In addition, skin Tregs preferentially express homing molecules such as CLA and CCR4, whereas central nervous system Tregs express CD103 and produce amphiregulin; both subsets contribute to tissue repair in their respective microenvironments (26). Single-cell RNA sequencing has revealed previously unrecognized Treg heterogeneity across tissues and disease states. For example, distinct Treg subpopulations exist in skin, gut, adipose, and CNS, each with unique transcriptomic signatures and functional specializations (27).

**Table 1:** Subsets of Tregs: origin, markers, tissue distribution, and functional characteristics.

Subset	Origin	Defining markers	Tissue distribution	Key functional characteristics
tTregs (thymus-derived)	Thymus	CD4^+^CD25^+^Foxp3^+^Helios^+^Nrp1^+^, TSDR hypomethylated	Secondary lymphoid organs, circulation, peripheral tissues	Stable Foxp3 expression, broad TCR repertoire, maintenance of peripheral tolerance
pTregs (peripherally derived)	Peripheral (TGF-β+retinoic acid)	CD4^+^Foxp3^+^Helios^-^Nrp1^-^, partial TSDR methylation	Mucosa (gut, lung), liver	Induced by tolerogenic DCs; important for mucosal tolerance
iTregs (*in vitro* induced)	*In vitro* (TGF-β+IL-2)	CD4^+^Foxp3^+^, variable Helios^-^	Not applicable (generated *ex vivo*)	Used for adoptive transfer therapy; may be less stable than tTregs
Adipose Tregs	tTregs or pTregs	PPARγ^+^ST2^+^CD44^+^	Visceral adipose tissue	Regulate adipose inflammation, insulin sensitivity
Intestinal Tregs	pTregs (predominant)	RORγt^+^GATA3^+^IL-10^+^	Lamina propria, intestinal lymphoid tissues	Suppress Th2/Th17 responses to commensal bacteria
Skin Tregs	tTregs	CLA^+^CCR4^+^CD103^+^	Dermis, epidermis	Promote wound healing, suppress skin inflammation
CNS Tregs	tTregs	CD103^+^amphiregulin^+^	Brain, spinal cord (under inflammation)	Limit neuroinflammation, promote tissue repair
Tumor-infiltrating Tregs	tTregs+pTregs	ICOS^+^PD-1^+^CCR8^+^CTLA-4^+^	Tumor microenvironment	Suppress anti-tumor immunity, promote immune evasion

TSDR, Treg-specific demethylated region; CNS, central nervous system; PPARγ, peroxisome proliferator-activated receptor γ; ST2, IL-33 receptor; RORγt, RAR-related orphan receptor gamma t; CLA, cutaneous lymphocyte antigen; CCR, C-C chemokine receptor; ICOS, inducible T-cell costimulator.

Stable expression of Treg signature genes, particularly Foxp3, is essential for maintaining Treg lineage identity and suppressive function. This stability is coordinately regulated by epigenetic mechanisms at three major levels. First, DNA methylation plays a central role. Treg signature genes such as Foxp3 and CTLA-4 contain TSDRs, and the hypomethylated state of the core TSDRs within the conserved non-coding sequence 2 (CNS2) region of the Foxp3 locus constitutes a key epigenetic hallmark that distinguishes Tregs from conventional T cells and ensures sustained Foxp3 expression (28, 29). Second, Tregs possess a distinctive histone modification landscape that regulates chromatin accessibility and acts in concert with DNA demethylation to preserve transcriptional stability and functional identity (30). Third, Treg-specific super-enhancers are activated early during Treg development and selectively control the expression of core lineage-defining genes, including Foxp3, thereby orchestrating Treg lineage commitment (31).

Despite differences in developmental origin and tissue localization, diverse Treg subsets generally exert immunosuppressive activity through similar mechanisms. These mechanisms can be broadly categorized into three major modes: metabolic competition, secretion of suppressive soluble factors, and cell contact-dependent inhibition, which collectively maintain peripheral immune tolerance.

With respect to metabolic competition, Tregs primarily consume IL-2 in the local microenvironment through high-level expression of CD25, thereby depriving effector T cells of a critical signal required for their activation and survival (32). In addition, Tregs express high levels of CD39 and CD73, which hydrolyze extracellular ATP into immunosuppressive adenosine; adenosine subsequently acts through the A2A receptor to inhibit T-cell activation (33).

In terms of soluble factor-mediated suppression, Tregs secrete multiple inhibitory cytokines, including IL-10, TGF-β, and IL-35. IL-10 suppresses the pro-inflammatory functions and antigen-presenting capacity of myeloid cells. TGF-β inhibits Th1 differentiation, restrains T-cell proliferation and CD8^+^ T-cell cytotoxicity, and contributes to the establishment of infectious tolerance. IL-35 can directly suppress effector T-cell function while promoting the expansion of iTregs (34).

CTLA-4 is centrally involved in contact-mediated immune suppression. Treg-specific deletion of CTLA-4 in mice results in spontaneous systemic lymphoproliferation, fatal T cell-mediated autoimmune disease, and excessive immunoglobulin E production, while simultaneously enhancing anti-tumor immunity (35). Moreover, selective CTLA-4 ablation markedly impairs the suppressive function of Tregs both *in vitro* and *in vivo*, particularly by weakening their ability to downregulate CD80 and CD86 expression on dendritic cells (DCs) (36). In addition, Tregs can directly induce target-cell lysis through granzyme A/B- and perforin-mediated cytotoxicity, thereby eliminating effector T cells, antigen-presenting cells (APCs), and natural killer (NK) cells. Notably, this cytotoxic activity is highly context-dependent; granzyme B expression and killing of NK cells and CD8^+^ T cells have mainly been observed in Tregs within the tumor microenvironment (37). Furthermore, Tregs can express high levels of LFA-1 and form stable interactions with DCs, thereby competitively preventing contact between effector T cells and DCs while also removing peptide–MHC complexes from the DC surface, ultimately attenuating antigen presentation (38).

## 3 Association of Treg abnormalities with autoimmune diseases

IPEX syndrome is an X-linked immunodeficiency disorder caused by mutations in the Foxp3 gene and is characterized by severe autoimmune manifestations, including type 1 diabetes (T1D), enteropathy, and eczema (39). Mutations in other Treg-associated signature genes can likewise give rise to severe autoimmune disease. For instance, individuals harboring mutations in CD25 may develop fatal autoimmune disorders (40). Autoimmune and inflammatory diseases have also been reported in families carrying heterozygous CTLA-4 mutations, which impair CTLA-4 expression in Tregs under both resting and activated conditions (41, 42). Lipopolysaccharide-responsive beige-like anchor protein (LRBA) is indispensable for CTLA-4 trafficking, and LRBA deficiency leads to loss of CTLA-4 expression in Tregs, thereby resulting in severe autoimmunity (43). In addition, monogenic disorders caused by mutations in BACH2 and STAT3 are also associated with Treg abnormalities. These mutations can induce enteropathy and other immune dysregulatory phenotypes, often accompanied by reduced numbers of circulating Tregs (44). Collectively, these findings provide compelling evidence that numerical deficiency or functional impairment of Tregs is closely linked to the pathogenesis of human autoimmune diseases. **Table 2** summarizes Treg abnormalities across major autoimmune diseases.

**Table 2:** Treg abnormalities in major autoimmune diseases.

Disease	Treg frequency (blood)	Treg frequency (inflamed tissue)	Suppressive function	Stability	Key references
Type 1 diabetes (T1D)	↓ (in new-onset) or ↔	↓ (pancreatic islets)	↓	↓ (ex-Tregs produce IFN-γ)	(45, 53)
Multiple sclerosis (MS)	↔ or ↓	↓ (CNS, during relapse)	↓	↓ (ex-Tregs produce IL-17)	(46, 53)
Systemic lupus erythematosus (SLE)	↓ (active) or ↔ (remission)	↑ (kidney, but dysfunctional)	↓ (CD25^+^ subset)	↓ (low Helios)	(47, 55, 56)
Rheumatoid arthritis (RA)	↔ or ↓	↑ (synovium, but impaired)	↓ (CTLA-4 low)	↓ (Foxp3 loss)	(50, 55)
Inflammatory bowel disease (IBD)	↔	↓ (gut lamina propria)	↓ (IL-10 low)	↓ (RORγt^+^ Treg conversion)	(52, 53)
Autoimmune uveitis	↔ or ↓	↓ (ocular tissues, during active)	↓	↓ (Th17-like conversion)	(51, 115)
Sjögren’s syndrome	↓	↓ (salivary glands)	↓	↓	(49)
Myasthenia gravis (MG)	↓ (thymoma-associated)	↔ (thymus)	↓	ND	(48)

↓, decreased; ↑, increased; ↔, unchanged; ND, not determined.

A large body of clinical evidence further indicates that defects in Treg number and/or function are present in the peripheral blood or inflamed tissues of patients with multiple autoimmune diseases, including T1D (45), multiple sclerosis (MS) (46), systemic lupus erythematosus (SLE) (47), myasthenia gravis (48), Sjögren’s syndrome (49), rheumatoid arthritis (RA) (50), autoimmune uveitis (51), and inflammatory bowel disease (IBD) (52). During disease progression, Tregs may exhibit impaired suppressive capacity, unstable Foxp3 expression, and production of pro-inflammatory cytokines such as IFN-γ and IL-17, thereby acquiring effector-like properties and exacerbating autoimmune tissue damage (53, 54). Nevertheless, some studies have reported that Treg frequencies in patients with SLE and RA are unchanged or even increased compared with healthy controls (55, 56).

Conflicting reports on Treg frequencies in diseases such as SLE and RA arise from multiple factors. Patient heterogeneity plays a major role: disease activity (active vs. remission), treatment status (naïve vs. on immunosuppressants), and disease duration all influence Treg numbers (56, 57). Methodological differences are equally important. Studies using CD25 as the sole marker may overestimate Tregs because activated effector T cells also upregulate CD25. In contrast, Foxp3-based identification offers higher specificity but requires consideration of its unstable expression under inflammatory conditions (58). The use of CD127 (low/negative) as a surrogate for Foxp3, or inclusion of Helios and TSDR methylation analysis, improves accuracy. Tissue compartment also matters: peripheral blood Treg frequencies often differ from those in inflamed tissues (e.g., synovium, kidney) (59). Meta-analyses have attempted to reconcile these discrepancies, confirming that stricter Treg definitions yield more consistent results across studies (60). Standardized marker panels and gating strategies are urgently needed for cross-study comparability.

Whether Treg abnormalities are primary or secondary varies by disease. In monogenic disorders (IPEX, CD25 deficiency, CTLA-4 haploinsufficiency), Treg defects are clearly primary (61). In multifactorial diseases (SLE, RA), both mechanisms coexist: genetic risk alleles predispose to Treg dysfunction, while chronic inflammation further impairs Treg stability, creating a vicious cycle (62). Treg abnormalities differ between systemic and organ-specific diseases. In SLE and RA, peripheral Treg numbers are often reduced, but inflamed tissues (kidney, synovium) may accumulate dysfunctional Tregs. In organ-specific diseases (T1D, MS, uveitis, IBD), Treg defects are more consistently observed within the target organ rather than in blood.

## 4 Preclinical studies of Treg-based therapy for autoimmune diseases

**Figure 2:**
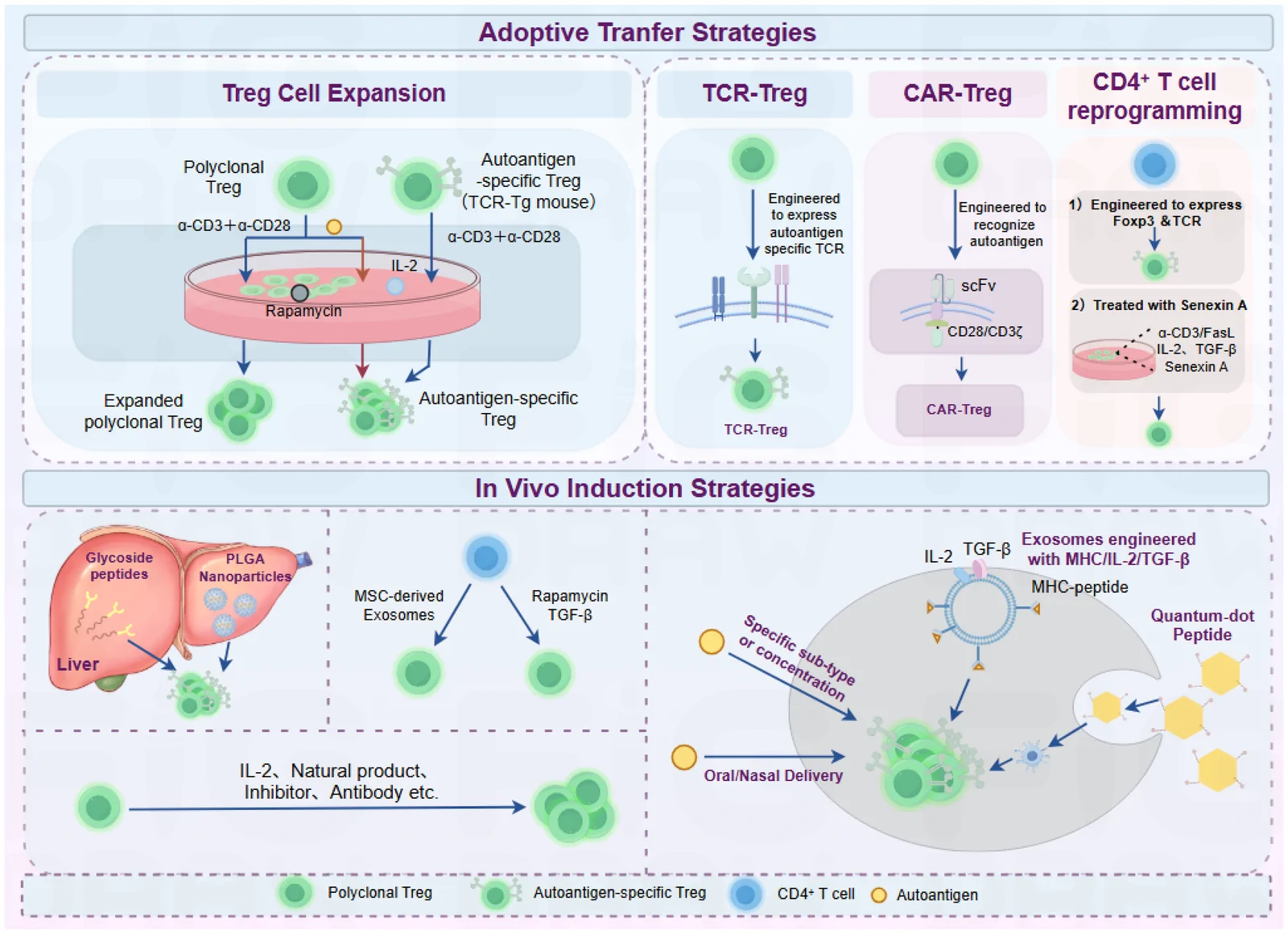
Treg-based immunotherapeutic strategies for the treatment of autoimmune diseases. This figure outlines two main strategies for Treg-based therapies in autoimmune diseases: adoptive transfer and *in vivo* induction. Under adoptive transfer, the top panel shows approaches to generate functional Tregs ex vivo: 1) Polyclonal Tregs are expanded using anti-CD3/CD28 beads with IL-2 and rapamycin. 2) Autoantigen-specific Tregs are generated either from TCR-transgenic mice or via genetic engineering to express antigen-specific TCRs (TCR-Tregs) or chimeric antigen receptors (CAR-Tregs). 3) Conventional CD4^+^ T cells can also be reprogrammed into Tregs through Foxp3 and TCR engineering or Senexin A treatment. The bottom panel illustrates *in vivo* induction strategies, including: 1) Liver-targeted delivery of glycoside peptides or PLGA nanoparticles to induce Tregs. 2) Use of MSC-derived exosomes, rapamycin, or TGF-β to promote Treg generation. 3) Oral/nasal delivery of specific autoantigens, 4) Exosomes engineered with MHC/IL-2/TGF-β, or 5) Quantum-dot peptides to induce antigen-specific Tregs. These diverse strategies aim to boost Treg numbers, enhance their antigen specificity, and restore immune tolerance in autoimmune diseases.

Given the indispensable regulatory role of Tregs in the progression of ADs, considerable efforts have been devoted to the development of Treg-based immunotherapeutic strategies. Essentially, current therapeutic strategies mainly aim to expand the number of Tregs (including polyclonal and antigen-specific Tregs), enhance their migratory capacity to inflammatory sites and immunosuppressive function, thereby restoring immune homeostasis and restraining excessive inflammatory responses (64, 73, 75, 80, 99, 137). **Figure 2** presents an overview of Treg-based immunotherapeutic strategies for the treatment of autoimmune diseases.

### 4.1 Adoptive transfer of Tregs

Infusion of exogenous Tregs is a cell-based immunomodulatory strategy centered on the isolation, purification, and directed *ex vivo* expansion of highly active and highly purified immunosuppressive cell populations, followed by reinfusion into animal models or patients after quality control assessment. By leveraging the intrinsic immunoregulatory properties of Tregs, this approach is designed to induce antigen-specific immune tolerance, rapidly correct immune disequilibrium, and re-establish immunological homeostasis. A large body of preclinical evidence has demonstrated the considerable therapeutic potential of adoptive Treg therapy in multiple autoimmune disorders, including inflammatory bowel disease (IBD), type 1 diabetes (T1D), experimental autoimmune encephalomyelitis (EAE), collagen-induced arthritis, and experimental autoimmune uveitis (EAU) (2). Below, we summarize five major research directions in this field.

#### 4.1.1 Therapy with polyclonal Tregs

Early studies on adoptive Treg therapy have extensively explored the therapeutic potential of polyclonal Tregs. The core procedure involves the isolation of Tregs followed by *ex vivo* expansion using anti-CD3/anti-CD28 antibodies in combination with IL-2, typically achieving a 300- to 500-fold expansion within 14 days. After expansion, the proportion of Foxp3^+^ Tregs generally exceeds 90%, and these cells express high levels of multiple phenotypic and functional markers, including CTLA-4, GITR, LAP, and CD25 (63). The addition of rapamycin during expansion helps preserve the immunosuppressive phenotype of Tregs and prevents their conversion into potentially harmful effector T cells (Teff), thereby improving the selective expansion of Tregs (64).

Extensive studies have shown that polyclonal Tregs are particularly effective in the prevention of ADs and can markedly suppress disease initiation (65, 66). However, their efficacy in individuals with established disease is more limited and appears to depend heavily on the route of administration. In EAU mice, intravitreal injection of polyclonal Tregs markedly reduced intraocular inflammatory infiltration and significantly improved clinical scores. This therapeutic effect was associated with reduced reactive oxygen species (ROS) production by infiltrating ocular cells, especially myeloid cells, as well as increased IL-10 production. By contrast, intravenous administration *via* the tail vein failed to produce comparable therapeutic benefit (67). Another study employed a physical mixture of hyaluronan and methylcellulose (HAMC) as an immune-protective and injectable hydrogel-based cell delivery system to improve the efficacy of Treg-based therapy for EAU. Intravitreal delivery of Treg-HAMC doubled the number of Treg trafficking to inflamed eyes in EAU mice and simultaneously enhanced their survival and stability. This system significantly reduced ocular inflammatory infiltrates, including IFN-γ^+^ and IL-17^+^ CD4 T cells, effectively alleviated ocular inflammation and preserved visual function. In contrast, intravitreal injection of Tregs without HAMC produced only marginal therapeutic effects in EAU (68). Adoptive transfer of polyclonal Tregs has also shown efficacy in rheumatoid arthritis (RA) and colitis models established in severe combined immunodeficient (SCID) mice and *Rag1^-/-^* mice (69, 70).

Nevertheless, the therapeutic efficacy of polyclonal Treg therapy remains constrained by several factors. A central limitation is the highly heterogeneous and stochastic nature of T-cell receptor (TCR) gene rearrangement in polyclonal Treg populations, resulting in an extremely low frequency of functional clones capable of specifically recognizing disease-relevant autoantigens. After infusion, in the absence of effective antigenic stimulation, most of these cells rapidly enter a quiescent state or undergo apoptosis, leading to an insufficient number of active suppressive cells capable of mediating durable immune regulation. Moreover, simply increasing the infusion dose of polyclonal Tregs to raise the absolute number of effective clones may readily induce systemic immunosuppression, thereby substantially increasing the risks of infection and potentially tumor development. Adoptively transferred polyclonal Tregs have a short half-life (71). Repeated dosing improves efficacy in preclinical models but increases manufacturing costs and infection risk. High-dose polyclonal Treg infusion may cause generalized immunosuppression, with increase in serious infections (72). This risk drives the development of antigen-specific Tregs.

#### 4.1.2 Therapy with *in vitro*-expanded antigen-specific Tregs

Compared with direct infusion of polyclonal Tregs, adoptive transfer of *in vitro* expanded antigen-specific Tregs requires a substantially lower number of cells and offers clear advantages in therapeutic efficacy. For instance, one study isolated and purified Tregs from BDC2.5 and GAD286 TCR transgenic (Tg) mice, followed by ex vivo expansion with anti-CD3, anti-CD28 and IL-2, which yielded approximately 200-fold expansion within two weeks. Notably, a small number of antigen-specific Tregs was sufficient to reverse T1D after disease onset (73). In another study, CD4^+^CD25^+^CD62L^+^ Tregs from BDC2.5 TCR Tg mice were expanded *in vitro* with cognate antigen peptide and IL-2 before transfer into NOD mice. A single infusion of 5 × 10^4^ expanded Tregs completely blocked the development of spontaneous diabetes before 13 weeks of age in NOD mice and effectively reversed hyperglycemia in 50% of mice with established diabetes (74).

#### 4.1.3 Therapy with TCR-Tregs

For autoimmune diseases in which pathogenic antigen-specific TCRs have been identified, it is theoretically feasible to generate TCR-engineered Tregs (TCR-Tregs) by introducing defined pathogenic antigen-specific TCRs into autologous or allogeneic Tregs using gene transfer technologies such as lentiviral transduction. This strategy endows Tregs with the capacity to precisely recognize disease-relevant antigens, thereby enabling targeted suppression of autoreactive lymphocyte activation while minimizing the systemic adverse effects associated with non-specific immunosuppression.

One preclinical study identified an HLA-DR15-restricted immunodominant T-cell epitope of the Smith (Sm) antigen through biophysical affinity-binding assays, followed by high-throughput single-cell sequencing to isolate the corresponding high-affinity Sm-specific TCR. Using a lentiviral vector, the selected Sm-specific TCR was transduced into peripheral blood-derived Tregs from anti-Sm antibody-positive, HLA-DR15-positive patients with lupus nephritis, thereby generating Smith antigen-specific-Tregs (Sm-Tregs). Compared with mock-transduced polyclonal Tregs, Sm-Tregs more effectively suppressed Sm-specific pro-inflammatory responses *in vitro* and inhibited disease progression in a humanized mouse model of lupus nephritis (75).

In another study focusing on anti-glomerular basement membrane (anti-GBM) disease, the non-collagenous domain 1 of the α3 chain of type IV collagen (Col4a3NC1), a core pathogenic autoantigen in anti-GBM disease, was used as the target. Immunodominant T-cell epitopes were first identified, followed by isolation of the corresponding antigen-specific TCRs. These TCRs were then introduced into CD4^+^CD25^+^ Tregs derived from healthy donors or patients. To preserve functional stability and suppressive capacity after induction, low-dose recombinant IL-2, TGF-β1, and rapamycin were used to prevent inflammatory reprogramming while maintaining stable Foxp3 expression. This protocol ultimately generated antigen-specific Tregs capable of recognizing Col4a3NC1 and effectively suppressing anti-GBM autoreactivity (76).

Moreover, in NOD mouse models of T1D, antigen-specific TCR-Tregs generated by viral transduction of TCRs derived from CD4^+^ T cells have been shown to effectively inhibit T effector cell proliferation and cytokine production (77, 78). In EAE, investigators retrovirally transduced human peripheral blood mononuclear cell (PBMC)-derived Tregs with a TCR recognizing myelin basic protein MBP_85–99_, thereby generating MBP_85–99_-specific TCR-Tregs. These cells infiltrated inflamed central nervous system tissues and ameliorated MOG-induced encephalomyelitis through bystander suppression (79).

#### 4.1.4 Therapy with CAR-Tregs

Chimeric antigen receptor-engineered Tregs (CAR-Tregs) are designed to confer antigen specificity on Tregs through genetic modification. In general, the procedure involves constructing a CAR directed against a target antigen. This receptor typically consists of an antigen-binding domain, an extracellular hinge region, a transmembrane domain, and intracellular signaling domains. The intracellular modules can be selected from molecules associated with Treg stability and suppressive function. The CAR-encoding sequence is then introduced into purified Tregs using an appropriate gene delivery platform to achieve stable and precise genetic integration. Through CAR-mediated recognition of autoantigens expressed at inflammatory sites, CAR-Tregs can achieve targeted migration and local retention within diseased tissues, thereby exerting site-specific immunosuppressive effects.

In 2008, Elinav and colleagues first proposed the concept of conferring antigen specificity on Tregs through CAR modification. Using 2,4,6-trinitrophenol (TNP), a colitis-associated antigen, they generated transgenic BALB/c mice expressing a tripartite chimeric receptor (TPCR) targeting TNP and subsequently isolated TNP-specific CAR-Tregs from these animals (80). TNP is not the only antigen used to investigate CAR-Treg therapy in murine IBD. Carcinoembryonic antigen (CEA), which is overexpressed in human colitis, has also been considered a potential target for CAR-Treg immunotherapy in IBD (81). CEA-specific CAR-Tregs maintained a functional Treg phenotype *in vitro*, including Foxp3 expression and IL-10 secretion, and also displayed preferential localization to the colon in murine colitis models (82).

To generate Tregs capable of recognizing islet antigens, one study engineered a CAR specific for the insulin B chain_10–23_ peptide presented by the I-Ag7 MHC class II allele unique to NOD mice (83). The InsBg7 CAR-Tregs not only suppressed BDC2.5 T cell-induced autoimmune diabetes by reducing the number of BDC2.5 effector T cells in peripheral lymphoid organs and the pancreas, but also prevented spontaneous autoimmune diabetes. By 30 weeks of age, 80% of mice in the control group had developed diabetes, whereas only 43% of mice receiving a single injection of InsBg7 CAR-Tregs became diabetic before 30 weeks. Similarly, GAD65-specific CAR-Tregs have also been developed for T1D (84), where they not only suppress pathogenic T cells but also outperform polyclonal Tregs in therapeutic efficacy.

Fransson and colleagues established an antigen-specific CAR-Treg induction strategy for EAE based on co-engineering of CAR and Foxp3. Specifically, myelin oligodendrocyte glycoprotein (MOG), a core pathogenic antigen in EAE, was selected as the target. The CAR construct included a Foxp3 co-expression element, with its extracellular domain containing an anti-MOG single-chain variable fragment for antigen recognition, a transmembrane region derived from CD28, and an intracellular domain combining the CD28 costimulatory domain and the CD3ζ signaling domain. The Foxp3 gene and CAR-coding sequence were assembled into the same lentiviral vector to achieve synchronized and stable expression of both CAR and Foxp3, thereby reinforcing suppressive function and phenotypic stability. Mouse naive CD4^+^ T cells were then infected with the lentiviral vector to generate MOG CAR-Tregs (85). For *in vivo* EAE treatment, only the CAR-Treg-treated group exhibited sustained improvement in clinical symptoms. By day 25, all mice in this group were asymptomatic. Following re-immunization on day 30, all control mice rapidly developed EAE symptoms within 2 days, whereas only one mouse in the CAR-Treg group developed mild symptoms (86). Of note, this type of CAR-Tregs also falls into the category of reprogramming CD4^+^ T cells into antigen-specific Tregs, which will be discussed in detail below.

Persistent ligand-independent basal CAR activation, known as tonic CAR signaling, constitutes an intrinsic layer of dysfunction encoded by CAR architecture and can compromise both the stability and immunosuppressive capacity of CAR-Tregs. It triggers profound metabolic reprogramming in Tregs featuring the disruption of oxidative phosphorylation (87, 88), upregulates exhaustion-associated transcription factors such as TOX and NR4A (87, 89), and compromises their competitive fitness against effector T cells for limited IL-2 within inflamed tissues (90). Collectively, these cascading defects markedly attenuate the *in vivo* therapeutic efficacy of CAR-Treg treatment for autoimmune disorders. Mitigation strategies include optimized scFv design, destabilizing domains, or inducible CAR systems (91). Off-target immunosuppression remains a critical safety concern for CAR-Treg therapy (7). Such unintended immune suppression can be mitigated through local delivery strategies (92), synthetic Notch (SynNotch) receptor engineering, or dual-antigen recognition designs (93). Overall, CAR-Tregs exhibit the greatest therapeutic potential for organ-specific autoimmune diseases with well-characterized autoantigens, including type 1 diabetes (94), uveitis (95), and anti-GBM disease (76).

On the other hand, the safety control mechanisms of CAR-Tregs also merit discussion. First, the iCasp9 suicide switch enables rapid and irreversible elimination of genetically engineered cells upon administration of AP1903. This mechanism has been clinically validated in hematopoietic stem cell transplantation and conventional CAR-T therapy, but its application in CAR-Tregs remains at the preclinical stage (96). Second, SynNotch receptors, upon recognition of a primary antigen, initiate the expression of CAR molecules or immunosuppressive effector molecules exclusively within the target tissue, thereby achieving precise spatial control (97). Inducible CAR expression systems are equally noteworthy, as they enable reversible and dose-titratable regulation of CAR expression (98). Collectively, these three strategies constitute complementary layers of a comprehensive safety architecture: iCasp9 serves as an ultimate “off switch,” SynNotch provides tissue-restricted targeting, and inducible systems afford dynamic modulation of activity. The rational combination of these technologies is the key to broadening the safety window of CAR-Tregs therapy.

CAR-Tregs and TCR-Tregs are the two most promising strategies with translational potential. However, they exhibit complementary differences across multiple key parameters (94). **Table 3** presents a systematic comparison of these approaches regarding antigen recognition, persistence, efficacy, safety, and manufacturing complexity.

**Table 3:** Comparison of CAR-Tregs and TCR-Tregs.

Parameter	CAR-Tregs	TCR-Tregs
Antigen recognition	MHC-independent (surface antigen)	MHC-restricted (peptide-MHC)
Targetable antigens	Cell surface proteins (e.g., insulin, GAD65, CEA)	Intracellular antigens (via MHC presentation)
TCR/promiscuity risk	Low (single-chain variable fragment)	Higher (potential mispairing with endogenous TCR)
Tonic signaling risk	Yes (can cause exhaustion)	Low
Off-target immunosuppression	Possible if antigen expressed on healthy tissues	Lower (MHC restriction limits off-target)
Manufacturing complexity	Moderate (lentiviral/retroviral transduction)	Higher (need to clone TCR α/β chains)
Preclinical efficacy	Strong in T1D, EAE, uveitis, colitis	Strong in T1D, EAE, lupus nephritis
Key advantages	MHC-independent, broader applicability	More physiological signaling, lower tonic activation
Key limitations	Tonic signaling, potential off-target effects	MHC restriction, TCR mispairing, patient-specific HLA matching

#### 4.1.5 Therapy with reprogrammed CD4^+^ T cells

In addition to directly engineering polyclonal Tregs *ex vivo* to confer antigen specificity, another highly promising strategy is to induce CD4^+^ T cells to differentiate into antigen-specific Tregs *in vitro*, followed by adoptive transfer.

GNTI-122, developed by the Rawlings laboratory, is one such cell-engineering platform. CD4^+^ T cells isolated from the peripheral blood of healthy donors or patients with T1D were engineered to stably express Foxp3 and an IGRP305-specific TCR recognizing an islet-specific peptide derived from islet-specific glucose-6-phosphatase catalytic subunit-related protein (IGRP). In addition, the engineered cells incorporated a chemically inducible signaling complex that responds to rapamycin to activate IL-2 signaling. The resulting engineered Tregs maintained a stable regulatory phenotype, were activated in an islet antigen-specific manner, and could suppress islet-specific effector T cells *in vitro* (99). For T1D treatment, these cells successfully prevented the development of hyperglycemia in T1D mice through day 70, whereas all control mice developed hyperglycemia by day 42. The group of Shimon Sakaguchi has also developed an innovative strategy for generating functionally stable iTregs from antigen-specific T cells. In this system, naive CD4^+^ T cells were stimulated with anti-CD3 antibody plus IL-2 and TGF-β. Additionally, the culture contained senexin A (a cyclin-dependent kinase 8/19 (CDK8/CDK19) inhibitor) and lacked CD28 co-stimulation, leading to high expression levels of Foxp3, CD25, and CTLA-4. A second stimulation under the original conditions with the addition of an anti-FasL antibody further increased the proportion of Foxp3^+^ cells to nearly 100% and also enhanced the mean fluorescence intensity of Foxp3, CD25, and CTLA-4. Importantly, sequencing analyses showed that the iTregs generated by this strategy closely resembled nTregs in both gene expression and epigenetic features, and they could be activated *in vivo* by cognate antigen (100, 101). The therapeutic efficacy of this strategy has also been well validated in murine models of IBD.

### 4.2 *In Vivo* induction strategies for expanding or enhancing endogenous Tregs

Enhancing the abundance or immunosuppressive activity of endogenous Tregs through inducers such as small-molecule agents, targeted biologics, or bioactive compounds derived from traditional Chinese medicine represents an immunomodulatory strategy with favorable safety and translational potential. Compared with exogenous cell infusion, this approach does not require *ex vivo* cell isolation, culture, or expansion, thereby avoiding the risk of immune rejection associated with cellular transplantation while also reducing manufacturing costs and technical complexity. Next, we summarize the currently strategies capable of expanding the pool of endogenous Tregs or enhancing their function.

#### 4.2.1 Cytokines

Since Tregs constitutively express abundant CD25 and outcompete effector T cells for IL-2 to restrict effector T cell proliferation and viability, numerous investigations have explored low-dose IL-2 as a strategy to selectively expand Tregs for autoimmune disease therapy. To date, low-dose IL-2 therapy has demonstrated promising efficacy in multiple disease models, including rheumatoid arthritis (RA) (102), type 1 diabetes (T1D) (103), experimental autoimmune encephalomyelitis (EAE) (104), and systemic lupus erythematosus (SLE) (105).

The therapeutic effects of low-dose IL-2 begin with controlled exposure of peripheral blood mononuclear cells (PBMCs) to IL-2, which initiates a cascade of intracellular signaling events predominantly through the Janus kinase/signal transducer and activator of transcription (JAK/STAT) pathway. Activation of this pathway ultimately upregulates Foxp3, and elevated Foxp3 expression not only reinforces the suppressive capacity of Tregs but also fine-tunes immune responses through regulation of numerous downstream pathways (106). At the same time, this signaling milieu activates additional transcription factors, including nuclear factor of activated T cells (NFAT), c-MYC, and STAT5A, which collectively shape the transcriptional and epigenetic landscape of Tregs, thereby strengthening their phenotypic stability and functional competence (107). During this process, effector memory T cells undergo only minimal upregulation, whereas Th1 cells are effectively restrained owing to reduced STAT4 activation. Consequently, low-dose IL-2 achieves relatively selective activation and expansion of Tregs, which are subsequently guided to inflammatory sites by homeostatic chemokines and cytokines. Importantly, low-dose IL-2 therapy not only increases Treg numbers but also enhances their suppressive function (108).

In addition, engineered IL-2 muteins and IL-2/anti-IL-2 immune complexes (IL-2–mAb complexes) have been developed to improve the preferential interaction of IL-2 with CD25, thereby enhancing selective Treg expansion and prolonging the half-life of IL-2. These optimized formulations can more effectively expand Tregs *in vivo* and improve therapeutic efficacy (104, 109).

TGF-β is another key cytokine that regulates Treg differentiation and functional maturation by inducing Foxp3 expression through activation of the SMAD signaling pathway (110). In addition, high-dose rapamycin promotes Treg expansion by inhibiting mTOR signaling and rebalancing the Treg/Th17 axis, thereby effectively delaying EAE progression and reducing clinical severity during the early, peak, and remission phases of disease (111). Moreover, combined administration of TGF-β and rapamycin can significantly enhance Treg expansion (112). To further optimize delivery, nanocarrier-based systems have been used to locally administer composite nanoparticles loaded with TGF-β, IL-2, and rapamycin *via* perilesional subcutaneous injection in models of dry eye disease and arthritis. This strategy enables *in situ* induction of Treg-mediated immunoregulation at inflammatory sites and provides a novel approach for the precise local intervention of autoimmune inflammation (113).

A systematic comparison of the three IL-2-based modalities reveals distinct profiles. Low-dose IL-2 has the most extensive clinical evidence. It selectively expands Tregs via high-affinity CD25 binding but has a short half-life (minutes to hours), requiring frequent injections. IL-2 muteins (e.g., IL-2v) reduce CD25 binding to minimize effector T cell activation, offering prolonged half-life and higher selectivity, but are at early clinical stages with higher costs. IL-2/anti-IL-2 complexes prolong half-life and potently expand Tregs in preclinical models, yet immunogenicity concerns limit clinical data. Overall, low-dose IL-2 currently has the strongest clinical support, while muteins and complexes represent promising next-generation options. To further minimize unintended Teff activation, ultra-low dosing, IL-2 muteins, or co-administration with rapamycin are effective strategies.

#### 4.2.2 Traditional Chinese medicine formulations and small molecules

A growing number of agents have been shown to ameliorate ADs progression by modulating the balance between Teff and Tregs. These include traditional Chinese medicine formulations and their active components, synthetic small molecules, and metabolites. **Table 4** provides a summary of small molecules and traditional Chinese medicine compounds that modulate Tregs in autoimmune diseases.

**Table 4:** Summary of small molecules and traditional Chinese medicine compounds that modulate Tregs in autoimmune diseases.

Compound/agent	Source/type	Mechanism of action (treg-related)	Functional outcome in AD models
Longdan Xiegan Decoction (LXD)	TCM formula	Upregulates Foxp3, increases IL-10/TGF-β, inhibits IL-6/STAT3	Ameliorates EAU; restores Th17/Treg balance
Kurarinone	Flavonoid from *Sophora flavescens*	Inhibits Rac1, prevents Treg-to-Th17 conversion	Improves EAU; outperforms prednisone
Sinomenine (SIN)	Alkaloid from *Sinomenium acutum*	Inhibits PI3K/AKT and NF-κB pathways	Restores Th17/Treg balance in EAU
Yerba Mate (YM)	Herbal infusion	Enhances Treg suppressive capacity	Attenuates EAE inflammation and demyelination
Upadacitinib	JAK1 inhibitor (synthetic)	Promotes Treg proliferation, inhibits CXCR4 migration	Ameliorates EAU
Methotrexate (MTX)	Antimetabolite	Increases Treg proportion in lymphoid tissues	Reduces inflammation in EAU
Itaconate (ITA)	Endogenous metabolite	Promotes Treg differentiation via DNAJA1/CDC45 axis	Corrects Teff/Treg imbalance in EAU
Progesterone (PRG)	Hormone	Upregulates TGF-β/IL-10 in Tregs, inhibits Th17	Improves retinal lesions in EAU
Melatonin	Endogenous hormone	Acts via ROS/TXNIP/HIF-1α; increases Treg proportion	Alleviates EAU
Ketogenic diet (KD)	Dietary intervention	Enhances TGF-β signaling, increases Treg frequency	Mitigates EAU severity

TCM, traditional Chinese medicine; EAU, experimental autoimmune uveitis; EAE, experimental autoimmune encephalomyelitis.

Natural products offer the advantage of relatively low toxicity and fewer adverse effects in disease treatment (114). In EAU rats, Longdan Xiegan Decoction (LXD) treatment significantly increases the number and proportion of CD4^+^CD25^+^Foxp3^+^ Tregs, upregulates Foxp3 expression, and enhances the immunosuppressive function of Tregs by elevating IL-10 and TGF-β secretion while restraining the IL-6/STAT3 pathway to stabilize Treg/Th17 immune homeostasis (115). Kurarinone improves EAU by increasing Foxp3 expression in Tregs, and inhibiting Rac1 activation, thereby suppressing excessive Th17-driven inflammation. Importantly, it also mitigates the conversion of Tregs toward a Th17-like phenotype, restores the Th17/Treg balance, and alleviates disease severity. Notably, kurarinone was found to outperform the conventional corticosteroid prednisone in restoring the Th17/Treg balance (116). Similarly, sinomenine (SIN) ameliorates inflammation in EAU rats by inhibiting activation of the PI3K/AKT and NF-κB pathways and restoring the Th17/Treg equilibrium (117). Another study found that Treg suppressive capacity was improved after *in vitro* Yerba Mate (YM) exposure. In EAU mice, YM treatment significantly increases Treg abundance and immune tolerance, while attenuating inflammation, immune cell infiltration and demyelination (118).

In EAU mice, upadacitinib not only promoted Treg proliferation but also suppressed aberrantly activated intercellular communication, particularly the CXCR4-mediated migratory pathway, which has been implicated in EAU pathogenesis (119).

Increasing evidence also supports the utility of antimetabolic drugs in Treg-targeted therapy for ADs. Methotrexate (MTX) and mycophenolate mofetil (MMF), both widely used as first-line immunomodulators in uveitis, have shown relevance in this context. Notably, MMF has recently been found to increase the proportion of Tregs in mesenteric lymph nodes and ocular tissues (120). Itaconate, an endogenous metabolite derived from the mitochondrial tricarboxylic acid (TCA) cycle, exerts favorable therapeutic effects in EAE by reducing Th17 differentiation and promoting Treg differentiation through metabolic and epigenetic reprogramming (121). In a study by Qi et al. (122), intraperitoneal administration of itaconate (ITA) produced stable and durable anti-inflammatory effects. Flow cytometric analysis showed that ITA reduced the proportions of Th1 and Th17 cells while increasing the proportion of Tregs in the lymph nodes of EAU mice. Mechanistically, the DNAJA1/CDC45 axis was identified as a key regulator of Teff/Treg imbalance in this setting. In addition, fluctuations in progesterone (PRG) levels have been reported to correlate with transient improvement or exacerbation in multiple autoimmune diseases. Interestingly, recent studies further suggest that PRG possesses immunomodulatory activity, as it can upregulate TGF-β and IL-10 expression in Tregs while suppressing pathological overactivation of Th17 cells, thereby correcting Th17/Treg imbalance and improving retinal lesions and inflammatory infiltration in EAU mice (123).

Melatonin, an endogenous hormone primarily synthesized by the pineal gland and retina and best known for regulating sleep and circadian rhythm, also exhibits antioxidant (124) and anti-inflammatory properties (125). In EAU mice, intraperitoneal melatonin administration markedly attenuated ocular inflammation, as evidenced by reduced optic disc edema, alleviated retinal vasculitis, and minimal retinal and choroidal infiltration. Mechanistic studies showed that melatonin acts through the ROS/TXNIP/HIF-1α pathway to suppress the transcription factors associated with peripheral Th1 and Th17 cells while increasing the proportion of Tregs (126).

In addition to circadian factors, dietary intervention also plays an important role in EAU. The ketogenic diet (KD), characterized by high fat and low carbohydrate intake, was originally introduced for refractory epilepsy because of its neuroprotective properties (127), but has recently emerged as a promising approach for immune-related disorders such as colitis (128). In EAU mice, KD increases the proportion of Tregs in lymph nodes, enhances TGF-β signaling, and mitigates disease severity (129).

#### 4.2.3 Antibodies

Monoclonal antibodies represent a central modality for precision treatment of ADs, primarily targeting key inflammatory pathways and pathogenic cell populations. A high-affinity monoclonal antibody against human SEMA5A, SYD12-12, has been generated and shown to suppress synovial hyperplasia and pannus formation while correcting Th17/Treg imbalance in collagen-induced arthritis (CIA) mice. Comparative studies indicated that SYD12–12 and adalimumab exerted similar therapeutic efficacy overall, although SYD12–12 was more effective at reducing IL-17A, whereas adalimumab showed superior suppression of TNF-α, IL-1β, and IL-6. Notably, combined use of the two agents produced significant synergistic effects (130). In addition, blockade of CD226, a T-cell costimulatory receptor associated with T1D risk, with a monoclonal antibody was shown to enhance the suppressive function of pancreatic and splenic Tregs in NOD mice (131). Anti-IL-6R monoclonal antibodies have likewise been shown in mouse models to induce Tregs while suppressing Th17 and/or Th1 differentiation (132).

Adeno-associated virus (AAV), a non-enveloped vector widely used in gene therapy because of its low pathogenicity and high transduction efficiency, can target specific retinal cell populations, display low immunogenicity, and support sustained transgene expression (133). In the context of EAU, AAV-based therapy can achieve prolonged therapeutic effects with fewer injections, demonstrating encouraging efficacy and safety and highlighting a potentially novel avenue for uveitis treatment (134). For example, one study employed an AAV2 system encoding the anti-TNF-α antibody adalimumab (ADA) to evaluate its efficacy in murine EAU. Administration of AAV2-ADA significantly reduced clinical, OCT, and histopathological scores, decreased the proportions of Th1 and Th17 cells in ocular tissues, and increased the proportion of Tregs in draining lymph nodes (135). By combining AAV vectors with monoclonal antibody-based strategies, this platform avoids the drawbacks of frequent dosing and may establish a durable immunosuppressive network through induction of regulatory populations such as Tregs, thereby reducing disease recurrence and offering a potential long-term solution for refractory or relapsing uveitis.

Antibody-based and cell-based Treg therapies exhibit distinct pros and cons. Antibody approaches are commercially available, cost-effective, and well-regulated, but require multiple administrations. In comparison, cell-based therapies can achieve durable immune tolerance after a single infusion, while their clinical application is hindered by complex manufacturing processes and unresolved safety concerns.

#### 4.2.4 Exosomes

Mesenchymal stem cells (MSCs) have been widely investigated in ADS therapy because of their potent immunosuppressive properties. Specifically, MSCs can enhance the suppressive function of Tregs. For example, MSC-derived indoleamine 2,3-dioxygenase (IDO) induces Foxp3 expression and increases splenic Treg frequencies in EAE mice, resulting in reduced clinical scores and disease severity (136). *In vitro*, co-culture of T cells with MSCs significantly upregulates Foxp3 expression in Tregs and increases the Treg proportion (137).

Exosomes are nanoscale membrane vesicles secreted by cells that naturally carry bioactive cargoes, including proteins, miRNAs, and lipids derived from their parent cells. They are now recognized as central mediators of intercellular communication and possess several attractive features, including high biocompatibility, low immunogenicity, and efficient transmembrane delivery capacity. As key mediators of MSC paracrine activity, MSC-derived exosomes (MSC-Exo) not only retain the robust immunoregulatory capacity of parental MSCs but also offer additional advantages such as lower immunogenicity, greater *in vivo* stability, and improved biosafety, thereby overcoming limitations associated with direct MSC administration, including immune rejection and poor engraftment efficiency (138). In EAU mouse models, adoptive transfer of MSCs (139), as well as genetically engineered MSC-Exo overexpressing CD73 (140) or IL-10 (141), has been shown to induce polyclonal Tregs, markedly alleviate ocular inflammation, and improve clinical manifestations. Our study also showed that MSCs mitigates EAU in an exosome-dependent manner and topical ophthalmic instillation of engineered MSC-Exo exosomes effectively ameliorate EAU (142).

In addition, Fang et al. engineered a fusion vesicle by loading the immunosuppressive agent CX5461 into grapefruit-derived nanovesicles and using gingiva-derived MSC-Exo as a CCR6 delivery platform. These fusion vesicles not only targeted inflamed sites with high CCL20 expression but also significantly increased splenic Treg frequencies in mouse models of psoriasis and atopic dermatitis, thereby providing a novel technical strategy for the treatment of cutaneous autoimmune and inflammatory disorders (143). Similarly, exosomes derived from human umbilical cord MSCs (hUMSCs) cultured in three-dimensional systems have shown strong immunomodulatory potential. These exosomes significantly increased Treg proportions in mice with vitiligo, promoted expression of suppressive mediators such as IL-10 and TGF-β, and effectively restored Treg suppressive function *in vitro*, suggesting their promise as an immunotherapeutic candidate for vitiligo (144).

Genetic engineering can further potentiate exosome efficacy. For example, Foxp1-overexpressing hUMSC-derived engineered exosomes markedly promoted Treg differentiation through the Foxp1/STAT5/Foxp3 signaling axis and exerted potent immunoregulatory effects in mouse models of SLE (145). Other studies have focused on exosome-mediated modulation of macrophage phenotype and the indirect induction of Tregs. In one such study, bone marrow stem cell-derived exosomes were used to treat renal macrophages from MRL/lpr mice, leading to marked upregulation of CD206, B7H4, CD138, Arg-1, CCL20, and anti-inflammatory cytokines, thereby promoting polarization toward an anti-inflammatory macrophage phenotype. These polarized macrophages subsequently recruited Tregs and effectively alleviated disease manifestations in MRL/lpr mice (146). In autoimmune hepatitis (AIH), engineered exosomes surface-modified with a CD4-targeting antibody and loaded with AAV6/Foxp3 specifically targeted the liver in concanavalin A-induced AIH mice, significantly increased hepatic Treg frequencies, and restored Treg-mediated immune tolerance (147).

Beyond the induction of polyclonal Tregs, an innovative strategy has recently been proposed in which engineered exosomes are combined with mTOR inhibition to induce antigen-specific Tregs. In this study, exosomes were engineered to co-display peptide–major histocompatibility complex class II (pMHCII), IL-2, and TGF-β on their surface, with these immunoregulatory molecules anchored via CD81 or milk fat globule-EGF factor 8 (MFG-E8), thereby generating engineered antigen-presenting extracellular vesicles (AP-EVs). *In vitro*, AP-EVs induced naive CD4^+^ T cells to differentiate into antigen-specific Tregs and promoted their proliferation as well as expression of regulatory markers including CD25, CTLA-4, PD-L1, and LAG-3. In 2D2 TCR transgenic mice, AP-EVs successfully induced the generation of MOG-specific Tregs. Further investigation showed that AP-EVs alone merely activated antigen-specific T cells *in vivo*, whereas co-administration of an mTOR inhibitor was required to drive differentiation toward the Treg lineage (148). Exosome-based therapies face batch-to-batch variability, lack of standardized isolation/characterization (MISEV guidelines), potency assay difficulties, and regulatory uncertainty for clinical-grade production.

#### 4.2.5 Concentration, delivery route, modification, and type of autoantigenic peptides

Administration of autoantigens does not necessarily elicit immune activation. Instead, they can induce immune tolerance under specific conditions. Among these variables, precise control of autoantigen concentration is a key determinant of the direction of the immune response. A substantial body of evidence has shown that autoantigens at low to intermediate physiological concentrations are preferentially taken up by tolerogenic dendritic cells (tDCs) in mucosal tissues, the liver, and secondary lymphoid organs. These DCs present antigen to naive CD4^+^ T cells through MHC class II molecules in a low-affinity yet persistent manner while simultaneously producing anti-inflammatory cytokines such as TGF-β and IL-10, thereby driving differentiation and stable expansion of antigen-specific Foxp3^+^ Tregs. By contrast, excessively high concentrations of autoantigens are more likely to activate inflammatory DCs, induce differentiation of pathogenic effector T cells such as Th1 and Th17 cells, and may even trigger systemic autoimmunity (149). For instance, continuous low-dose subcutaneous administration of the InsB_9–23_ peptide significantly induced insulin-specific Tregs in pancreatic draining lymph nodes and effectively delayed and prevented T1D onset in NOD-SCID mice, whereas a single high-dose injection of the same peptide accelerated disease development (150).

Among delivery strategies, oral and mucosal administration are well-established routes for tolerance induction. As the largest immune barrier in the body, the mucosal immune system provides a unique microenvironment that helps prevent systemic inflammatory responses to antigen exposure. Autoantigens delivered via oral or mucosal routes can be selectively captured by DCs in mucosa-associated lymphoid tissues, many of which exhibit a tolerogenic phenotype and secrete anti-inflammatory cytokines such as TGF-β and IL-10. These signals promote the differentiation of naive T cells into antigen-specific Tregs and thereby support long-term maintenance of immune tolerance (151–153). In addition, modification of antigens with N-acetylgalactosamine or N-acetylglucosamine enables intravenous targeting of hepatic antigen-presenting cells, allowing the unique tolerogenic liver microenvironment to enhance antigen presentation and induce antigen-specific Treg generation. This antigen-modification strategy offers several advantages: it not only effectively prevents T1D (154), but can also induce tolerance in the setting of an established immune response, with cross-species validation in both murine EAE and non-human primate SIV vaccine response models (155).

Nanoparticles, owing to their favorable biocompatibility, biodegradability, and low toxicity, have been widely explored as carriers for antigen delivery to induce antigen-specific Tregs and restore immune tolerance. For example, in EAE mouse models (156, 157), intravenous administration of PLGA particles surface-coupled with the myelin peptide PLP_139–151_ resulted in uptake by splenic and hepatic antigen-presenting cells, peptide processing and presentation via MHC class II molecules, and subsequent induction of T-cell anergy together with Treg expansion. In another study, biodegradable microparticles (MPs) were designed to carry both MHC class II/PLP complexes and cargoes containing rapamycin together with an IL-2 fusion protein. This strategy reversed disease in 100% of symptomatic EAE mice, and 38% of mice achieved complete recovery (158). Combined administration of rapamycin, IL-2 protein, and target antigen has also been validated in preventive models of T1D and primary biliary cholangitis (159), where it significantly and synergistically increased antigen-specific Tregs *in vivo*. Ionizable lipid nanoparticles can also serve as carriers for non-inflammatory mRNA vaccines encoding autoantigens. These vaccines target secondary lymphoid organs without triggering innate inflammatory responses, thereby efficiently presenting autoantigens within a tolerogenic immune microenvironment and selectively inducing and expanding immunosuppressive antigen-specific Tregs. In EAE, this strategy markedly suppressed activation and infiltration of pathogenic autoreactive effector cells such as Th1 and Th17 cells, restored central nervous system and peripheral immune tolerance, and ultimately alleviated clinical disease (160).

Quantum dots (QDs), a novel class of fluorescent nanomaterials characterized by uniform size, excellent optical properties, and facile surface modification, have also shown unique potential in autoantigen delivery and Treg induction. For example, subcutaneous administration of QDs surface-modified with MOG_35–55_ peptide led to rapid accumulation in draining lymph nodes, where they were taken up by macrophages and DCs through scavenger receptors. This uptake promoted the conversion of APCs toward a tolerogenic phenotype and enhanced antigen presentation, thereby driving robust expansion of MOG-specific Tregs. This strategy reduced EAE incidence by approximately 10-fold, significantly lowered clinical scores, and did not produce obvious systemic toxicity (161). In addition, liposomes co-loaded with MOG_35–55_ peptide and an aryl hydrocarbon receptor (AhR) agonist induced DCs to acquire a tolerogenic PD-L1^high^ phenotype while presenting antigen, thereby expanding MOG-specific Tregs and Tr1 cells and reducing Teff infiltration into the central nervous system, ultimately leading to marked attenuation of CNS inflammation (162).

Importantly, many autoantigenic peptides are intrinsically biased toward induction of antigen-specific immune tolerance. Unlike conventional strategies that rely primarily on delivery of antigen within a tolerogenic environment, the biochemical properties of these peptides themselves contribute to their tolerogenic potential. First, these autoantigenic peptides generally bind MHC molecules with relatively low affinity, and the resulting peptide–MHC complexes dissociate rapidly. As a consequence, they deliver only transient and low-intensity TCR signals that fail to reach the threshold required for full T-cell activation, thereby preferentially inducing clonal anergy, clonal deletion, or Treg differentiation rather than pathogenic Th1/Th17-type inflammatory responses (163). Second, many such peptides function as weak agonists or partial agonists. Their intrinsic TCR-contact residues induce only incomplete conformational changes in the TCR, resulting in signaling bias: pro-inflammatory pathways such as NF-κB and MAPK are not efficiently activated, whereas negative regulatory molecules such as Cbl-b and SHIP-1 are selectively upregulated, promoting secretion of immunosuppressive cytokines including IL-10 and TGF-β (164).

On the basis of these principles, altered peptide ligand (APL) technology has emerged as a core strategy for the rational design of antigen-specific tolerogens. APLs are generated by introducing site-directed mutations into key amino acid residues of natural autoantigen peptides, thereby selectively reducing TCR agonistic activity while preserving MHC-binding capacity. In this way, a strongly pathogenic agonistic peptide can be converted into a therapeutically useful tolerogenic ligand (165). This strategy has achieved remarkable efficacy in multiple animal models of autoimmune disease. For example, a single amino acid mutant of PL_139-151_ (substitution of tryptophan at position 144 with glutamine) was shown to promote secretion of immunoregulatory cytokines such as IL-4 and IL-10, induce Th2 immune deviation and antigen-specific Treg differentiation, and completely prevent EAE (166). In collagen-induced arthritis (CIA) mice, intranasal administration of a mutated influenza hemagglutinin peptide, HA_308-317_, significantly increased Treg numbers in the spleen and draining lymph nodes and enhanced their suppressive function, producing superior therapeutic effects compared with the wild-type HA_308–317_ peptide (167).

## 5 Clinical translation of Treg-based therapy for autoimmune diseases

At present, clinical trials of Treg-based therapy for ADs mainly focus on two core strategies: adoptive Treg transfer therapy and low-dose IL-2 treatment. According to data from ClinicalTrials.gov, 37 relevant clinical trials have been registered to date, with type 1 diabetes (T1D), multiple sclerosis (MS), and rheumatoid arthritis (RA) as the primary disease indications.

From the perspective of treatment modalities, adoptive Treg transfer therapy encompasses three major technical approaches: autologous peripheral blood-derived polyclonal Tregs, umbilical cord blood-derived polyclonal Tregs, and genetically engineered Tregs. Meanwhile, low-dose IL-2-based therapy has been evaluated under three primary clinical modalities: monotherapy, combination regimens, and modified IL-2 formulations. Beyond these two core strategies, a small number of clinical studies have also investigated monoclonal antibody monotherapy, as well as combination regimens combining adoptive Treg transfer with monoclonal antibodies.

Of 37 registered trials, 13 are completed and 7 have published efficacy results. Most show safety but variable efficacy. Strongest signals are in T1D (low-dose IL-2), SLE (low-dose IL-2), and GVHD (Treg infusion). Slow progress is due to high costs and complexity of Treg manufacturing, lack of potency assays, poor Treg persistence, and regulatory uncertainty.

Nevertheless, due to hurdles including difficult patient recruitment and lengthy study timelines, the outcomes of many relevant clinical trials remain unpublished. Furthermore, the long-term efficacy and safety of Treg-based therapies for ADs still warrant further validation in clinical studies with larger sample sizes and extended follow-up durations. **Table 5** summarizes current clinical studies of Treg-based therapies, detailing the enrolled autoimmune diseases, therapeutic regimens, trial identifiers, research progress, sample sizes, primary endpoints, clinical efficacy outcomes, and safety profiles.

**Table 5:** Clinical trials of Treg-based therapy for autoimmune diseases.

Disease	Therapeutic approach	Trial ID	Phase	n	Primary endpoint	Efficacy outcome	Safety
Type 1 diabetes (T1D)	CD6-CAR-Tregs	NCT07395050	I	6	Safety	Ongoing	Ongoing
Type 1 diabetes (T1D)	Autologous polyclonal Tregs	NCT03162237	NA	20	Blood sugar stability	The clinical efficacy is good	No SAEs
Type 1 diabetes (T1D)	Tregs+anti-CD20	NCT06688331	II	150	Feasibility	Ongoing	Ongoing
Type 1 diabetes (T1D)	Autologous expanded Tregs	NCT01210664	I	16	Safety	Treg persist; maintain C-peptide levels	No SAEs
Type 1 diabetes (T1D)	Autologous Tregs+IL-2	NCT02772679	I	16	Safety; Treg lifespan	Partial metabolic improvement	No SAEs
Type 1 diabetes (T1D)	Autologous Tregs	NCT06708780	I	20	Safety	Ongoing	Ongoing
Type 1 diabetes (T1D)	TCR-Tregs	NCT06427421	NA	80	Feasibility	NA	NA
Type 1 diabetes (T1D)	Bispecific antibody (KIR/CD8)	NCT06324604	I	96	Safety and tolerability	Expand CD8 Tregs; increase C-peptide levels	No SAEs
Type 1 diabetes (T1D)	Low-dose IL-2	NCT02411253	II	141	Efficacy assessment	NA	NA
Type 1 diabetes (T1D)	Umbilical cord Tregs+liraglutide	NCT03011021	I/II	40	Safety	NA	NA
Type 1 diabetes (T1D)	Umbilical cord Tregs	NCT02932826	I/II	40	Safety	Metabolic improvement	NA
Autoimmune hepatitis	Autologous Tregs	NCT02704338	I/II	30	Immunological remission; Biochemical remission	NA	NA
Ulcerative colitis	Autologous Tregs	NCT04691232	I	11	Safety and tolerability	NA	NA
Rheumatoid arthritis	Tocilizumab (anti-IL-6)	NCT02963402	NA	35	Changes in Treg phenotype	NA	NA
Rheumatoid arthritis	CAR-Tregs (CD19)	NCT06201416	I	24	Safety	Clinical response rate (83.3%)	No SAEs
Multiple sclerosis	TCR-Tregs	NCT06566261	I	12	Safety and tolerability	Ongoing	Ongoing
Multiple sclerosis	Oral nanocurcumin	NCT03150966	II	41	EDSS score	NA	No SAEs
MS&RA	CAR-Tregs	NCT07502859	I	24	Safety and tolerability	Ongoing	Ongoing
IPEX	Engineered autologous Tregs	NCT05241444	I	30	Safety and Feasibility	Ongoing	Ongoing
Multiple ADs	Low-dose IL-2	NCT01988506	II	81	Changes in peripheral blood Treg proportion	Treg expansion; B cell reduction; clinical improvement.	Mild to moderate AE, no SAE or DLT
dcSSc&RA	CD19 CAR-Tregs	NCT07473154	I/II	16	Safety and tolerability	Ongoing	Ongoing
Hidradenitis suppurativa	CAR-Tregs	NCT06361836	I	24	Safety	Ongoing	Ongoing

SAEs, serious adverse events; AEs, adverse events; NA, not available; ADs, autoimmune diseases; IPEX, immune dysregulation, polyendocrinopathy, enteropathy, X-linked syndrome; dcSSc, diffuse cutaneous systemic sclerosis.

## 6 Current challenges of Treg-based immunotherapy for ADs

Despite a series of important advances in Treg-based therapy for ADs, its translation into standardized clinical application still faces multiple challenges, which severely limit the full realization of its therapeutic potential.

### 6.1 Challenges related to the biological characteristics of Tregs

At present, the vast majority of clinical trials still rely on the adoptive transfer of polyclonal Tregs. A major challenge of polyclonal Treg-based therapy lies in obtaining a sufficient number of cells with stable phenotype and function. nTregs account for only approximately 1%–2% of peripheral blood CD4^+^ T cells, making their isolation inherently difficult. In addition, these cells exhibit substantial phenotypic heterogeneity and variable suppressive activity. More importantly, autologous Tregs derived from patients with ADs often display reduced cell numbers, impaired suppressive function, abnormal epigenetic modifications, and unstable Foxp3 expression (168). The core mechanisms underlying Treg instability mainly include: (1) epigenetic silencing of Foxp3 via CNS2 hypermethylation; (2) loss of Helios; (3) conversion to IFN-γ^+^ or IL-17^+^ ex-Tregs. The major predictive biomarkers for Treg instability consist of TSDR methylation level, Helios expression, and CD45RA phenotype (169, 170).

During *ex vivo* culture, even when stability-promoting agents such as rapamycin and IL-2 are included, prolonged expansion cannot fully prevent phenotypic drift of Tregs. This is typically manifested by downregulation of inhibitory surface molecules, including CD25 and CTLA-4, as well as reduced secretion of immunosuppressive cytokines such as IL-10 and TGF-β, thereby compromising the functional uniformity of therapeutic Treg products (171). Several strategies have been explored to enhance Treg stability and suppressive capacity, including CRISPR-Cas9-mediated stabilization of Foxp3 and Helios expression, epigenetic editing of Foxp3, prevention of Foxp3 polyubiquitination, and RNA interference targeting protein kinase C θ (PKCθ). However, these approaches do not fundamentally overcome the intrinsic non-specificity of polyclonal Tregs or the limited efficacy associated with this therapeutic modality (172). Following reinfusion, Tregs generally exhibit a short *in vivo* half-life, typically ranging from only a few days to several weeks. Moreover, due to insufficient survival signals, they are readily eliminated by the host immune system and often fail to efficiently engraft within inflamed tissues, making it challenging for a single therapy to yield long-lasting therapeutic benefits. Even more concerning, the inflammatory microenvironment may drive phenotypic instability and promote the conversion of Tregs into pro-inflammatory Teff, potentially exacerbating autoimmune inflammation. Strategies to maintain Foxp3 expression (CRISPR stabilization, epigenetic editing, rapamycin culture) are effective *in vitro* but lack robust *in vivo* validation. Further studies are needed before clinical application (173).

### 6.2 Challenges related to targeted delivery

Polyclonal Tregs, which are widely used in clinical studies, lack the ability to specifically recognize ADS-related autoantigens. As a result, they tend to distribute diffusely *in vivo*, making it difficult to achieve effective enrichment within diseased tissues. Moreover, they face additional barriers in penetrating tissue-restricted sites such as the blood–brain barrier, synovium, and pancreatic islets, and are therefore unable to selectively eliminate pathogenic autoreactive T-cell clones (174). To address these limitations in targeting specificity, several strategies have emerged, including CAR-Tregs, TCR-Tregs, and the induction and expansion of antigen-specific Tregs using disease-relevant autoantigens. Nevertheless, these approaches continue to face substantial challenges. TCR-Tregs are mainly constrained by MHC restriction and potential mispairing with endogenous TCR chains (94). Although CAR-Tregs can overcome MHC dependency, many autoantigens are expressed not only at sites of autoimmune inflammation but also in healthy tissues elsewhere in the body. This raises a significant risk of off-target immunosuppression, which remains difficult to avoid (80). In addition, persistent CAR signaling may compromise Treg stability and accelerate functional exhaustion. Genetic engineering of chemokine receptors efficiently enhances the tissue-specific homing capacity of Tregs. Specifically, CXCR3 engineering enables Treg recruitment to CXCL10-expressing pancreatic tissues for type 1 diabetes (175), CCR4/CCR6 potentiates skin targeting (176), CCR9 facilitates gut tropism (177), and CXCR4 confers central nervous system (CNS) infiltration (178). In addition, SynNotch receptors support precise, multi-step targeting of pathological tissues. More importantly, most ADs are characterized by multifactorial pathogenesis and the involvement of multiple autoantigens, such that Tregs directed against a single antigen are unlikely to cover the full spectrum of pathogenic autoreactive clones or achieve comprehensive restoration of immune tolerance. To make matters worse, the pathogenic antigens involved in many organ-specific ADs remain incompletely defined. Some autoantigens also share sequence homology with tumor-associated antigens, raising the possibility that antigen-specific Tregs may inadvertently suppress antitumor immunity through cross-reactivity, thereby increasing the potential risk of tumor development. For autoimmune diseases characterized by epitope spreading (e.g., T1D, MS), multiple therapeutic strategies are available, including the combination of distinct CAR-Treg populations, the adoption of multi-specific CARs, and targeted modulation of shared autoantigens. Despite their relatively lower antigen specificity, polyclonal Tregs may be better suited for treating such diseases (179).

### 6.3 Challenges related to manufacturing technologies

GMP-compliant Treg manufacturing mandates strict quality benchmarks: cell viability ≥ 85%, Foxp3^+^ purity ≥ 70%, and sustained Foxp3 positivity (≥ 80%) after thawing (180). Achieving scalable clinical production necessitates closed automated culture systems and universal off-the-shelf allogeneic Treg platforms. However, current approaches for Treg manufacturing still lack standardized protocols for cell isolation, expansion, and quality control, resulting in substantial product heterogeneity. In addition, existing expansion systems are prone to contamination by pro-inflammatory Teff, leading to high impurity rates. Cryopreservation and subsequent thawing may further cause significant declines in cell viability, phenotypic stability, and suppressive function. On the other hand, different gene-modification strategies are associated with complex procedures, prolonged production cycles, and high costs. Moreover, viral vectors commonly used in engineered Treg generation carry risks of insertional mutagenesis, genotoxicity, and immunogenicity. Although CRISPR-based technologies offer promising opportunities for precise editing, their relatively limited efficiency and the potential for off-target effects remain important issues to be resolved.

### 6.4 Challenges related to safety and clinical translation

From a safety perspective, the infusion of high doses of non-specific Tregs may induce systemic immunosuppression, thereby increasing potential risks such as infection and tumor development. Meta-analyses show ~1.8% infection rate with Tregs (181). No clear malignancy increases with 1–5 years follow-up (182). Long-term data from T1D patients show sustained tolerance up to 5 years in some cases (183). In addition, genetically engineered Tregs carry risks related to insertional mutagenesis, oncogenic transformation, and off-target genome editing. Furthermore, the long-term risks associated with repeated or prolonged Treg infusion have not yet been adequately evaluated.

Clinical translation is further complicated by the fact that the mechanisms underlying Treg dysfunction vary across different ADs, making it difficult for any single therapeutic strategy to be broadly applicable. As a result, disease-specific Treg-based therapeutic approaches remain insufficiently developed. Moreover, most existing clinical trials are limited by small sample sizes and short follow-up durations, and there is still a lack of large-scale, long-term clinical data to define the optimal therapeutic regimen for Treg infusion. Patient responses also show marked interindividual variability: while some patients achieve favorable therapeutic outcomes, others exhibit minimal response or even develop treatment resistance. A central explanation for this phenomenon is the intrinsic heterogeneity of patient responsiveness. However, effective biomarkers for predicting therapeutic responses and guiding treatment optimization remain lacking, posing a major obstacle to the clinical implementation of personalized Treg-based therapy. At the same time, there is currently no unified quality control framework for Treg products, including standardized criteria for cell purity, viability, suppressive function, phenotypic stability, and pathogen testing. This lack of harmonized quality standards makes it difficult to reliably ensure product quality and safety, further impeding the clinical translation of Treg-based therapies.

On the other hand, *in vivo* interventions designed to induce Treg formation or boost Treg function carry a risk of severe adverse reactions. For example, while *in vivo* administration of immunosuppressive agents efficiently induces Foxp3^+^ Treg differentiation and enhances immune tolerance, their clinical application is limited by adverse side effects, including accelerated tumor growth and metastasis and elevated susceptibility to infection due to suppressed normal immune defense.

For therapeutic response prediction, a set of reliable biomarkers has been established to differentiate responders from non-responders to Treg therapy, paired with standardized monitoring protocols to improve clinical safety. Primary predictive biomarkers consist of baseline Treg frequency (<2% of total CD4^+^ T cells), TSDR hypomethylation, and diminished serum IL-2 concentrations. Longitudinal clinical monitoring involves serial Treg immunophenotyping for Foxp3 and Helios, functional suppression assays, and routine viral load monitoring for CMV and EBV.

## 7 Future perspectives

The Treg therapy field is advancing rapidly through emerging technologies across four directions: (1) CRISPR-engineered Tregs. Genome-wide CRISPR screening has identified RBPJ as a key negative regulator of Foxp3 induction in human iTregs; RBPJ knockout enhances iTregs differentiation, phenotypic stability via CNS2 demethylation, and suppressive function in xenogeneic GVHD models (184). In addition to direct editing of Tregs, CRISPR enables off-the-shelf allogeneic Tregs: non-viral editing disrupting B2M and CIITA while inserting HLA-E-B2M fusion creates hypo-immunogenic Tregs that evade T and NK cell attack and prolong xenograft survival in humanized mouse models (185). These advances support ready-to-use Treg products that overcome donor variability and manufacturing delays. (2) Synthetic biology approaches. synNotch receptors engineered into conventional CD4^+^ T cells enable antigen-triggered production of anti-inflammatory payloads (IL-10, TGF-β1, PD-L1) along with IL-2 sinks (CD25). Such synthetic suppressor T cells protected transplanted beta cell organoids and specific tissues from CAR T cell cross-reaction without systemic immunosuppression (93). SynNotch transcriptional circuits enable location-specific payload expression, achieving better efficacy than anticipated with no off-target toxicity (186). Logic-gated CARs, split-CAR systems, and inducible safety switches (e.g., iCasp9) further enhance spatial control. (3) Next-generation CAR-Treg platforms. “Armored” CAR-Tregs represent a major advance. CRISPR-mediated PD1 deletion combined with IL-10 knock-in under the PD1 promoter creates CAR-induced IL-10 secretion. These hybrid CAR-Tregs suppress dendritic cells and autoantigen-specific T cells more potently than conventional CAR-Tregs, demonstrating that simultaneous removal of inhibitory signals and enhancement of suppressive mechanisms improves potency (187). Fourth-generation CAR-Tregs secreting IL-10 or TGF-β upon activation, dual-antigen CARs for epitope spreading, and cytokine receptor-armored CARs that expand without lymphodepletion are under development. (4) iPSC-derived Tregs. Induced pluripotent stem cells offer a renewable, standardized source for Treg generation. Collaborative studies demonstrate that iPSC-derived cells can acquire key regulatory T-cell characteristics while remaining compatible with scalable production, supporting off-the-shelf allogeneic Treg therapies for autoimmune diseases (188). Key challenges include maintaining FOXP3 stability during expansion and achieving sufficient yields, but progress in differentiation protocols is narrowing the gap toward clinical feasibility.

Together, these technologies are shifting Treg-based therapy from broad immunosuppression toward precise, durable immune tolerance. With continued advances, they hold the potential to achieve long-term remission, and possibly cure, for a wide range of autoimmune diseases.
